# Back to Pupillometry: How Cortical Network State Fluctuations Tracked by Pupil Dynamics Could Explain Neural Signal Variability in Human Cognitive Neuroscience

**DOI:** 10.1523/ENEURO.0293-16.2017

**Published:** 2017-12-26

**Authors:** Miriam Schwalm, Eduardo Rosales Jubal

**Affiliations:** 1 Focus Program Translational Neuroscience (FTN) and Institute for Microscopic Anatomy and Neurobiology, Johannes Gutenberg-University Mainz, Mainz D-55128, Germany; 2 GRADE Brain, Goethe Graduate Academy and FB 15, Goethe University Frankfurt, Frankfurt D-60438, Germany; 3 Instituto de Ciencias Biomédicas, Universidad Autónoma de Chile, Santiago, Chile

**Keywords:** network state changes, pupil diameter, pupillometry

## Abstract

The mammalian thalamocortical system generates intrinsic activity reflecting different states of excitability, arising from changes in the membrane potentials of underlying neuronal networks. Fluctuations between these states occur spontaneously, regularly, and frequently throughout awake periods and influence stimulus encoding, information processing, and neuronal and behavioral responses. Changes of pupil size have recently been identified as a reliable marker of underlying neuronal membrane potential and thus can encode associated network state changes in rodent cortex. This suggests that pupillometry, a ubiquitous measure of pupil dilation in cognitive neuroscience, could be used as an index for network state fluctuations also for human brain signals. Considering this variable may explain task-independent variance in neuronal and behavioral signals that were previously disregarded as noise.

## Visual Abstract

**Figure:**
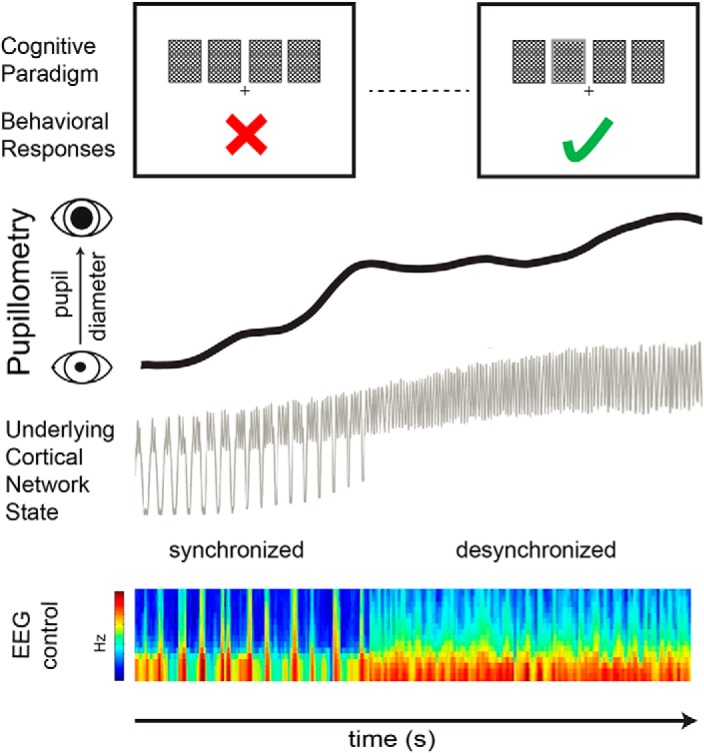

## Significance Statement

The mammalian thalamocortical system generates intrinsic activity reflecting different states of excitability, arising from changes in the membrane potentials of underlying neuronal networks. Fluctuations between these states occur spontaneously, regularly, and frequently throughout awake periods and influence stimulus encoding, information processing, and neuronal and behavioral responses. Changes of pupil size have recently been identified as a reliable marker of underlying neuronal membrane potential and thus can encode associated network state changes in rodent cortex. This suggests that pupillometry, a ubiquitous measure of pupil dilation in cognitive neuroscience, could be used as an index for network state fluctuations also for human brain signals. Considering this variable may explain task-independent variance in neuronal and behavioral signals that were previously disregarded as noise.

## 

Neural networks undergo constant state fluctuations provoked by changes of internally generated activity, even in the absence of external stimulation. These changing patterns of activity vary on slow and rapid time scales and shape the ongoing signal as well as neuronal responses on incoming sensory information.

At the behavioral level, these states can be associated with different levels of arousal and attention, or other nonsensory signals directly related to behavioral performance (Hall et al., 2014; Reimer et al., 2014). For example, alternating states of excitability are associated with changes in global network activity, as between sleep and wakefulness (Steriade et al., 2001; Destexhe et al., 2007), from inattention to vigilance (Boly et al., 2007; McGinley et al., 2015a) or from resting to locomotion (Poulet and Petersen, 2008; Eggermann et al., 2014).

Network states provide a rich experimental variable, which can explain a multitude of neuronal response properties. In rodents, sensory responses have been found to be noticeably shaped by the dominant network activity pattern (Gao et al., 2009; Stroh et al., 2013; Schwalm et al., 2017). Specifically, neuronal responses can be altered in magnitude and signal-to-noise ratio (McGinley et al., 2015a), latency (Lima et al., 2011; Vinck et al., 2015; Schwalm et al., 2017), neuronal variability (Zagha et al., 2013; McGinley et al., 2015a; Schölvinck et al., 2015), and noise correlations (Poulet and Petersen, 2008; Vinck et al., 2015). Studies using macroscopic measures of ongoing signals as resting state functional magnetic resonance imaging (rs-fMRI) measurements usually reveal transient changes in brain activity related to vigilance drifts, complicating the analysis of such data (Schneider et al., 2016). Those changes in the rs-fMRI signal were shown to correspond to fluctuations of intracortical electrophysiological recordings while tracking behaviorally relevant arousal changes in macaques (Chang et al., 2016). In humans, functional connectivity has been linked to global state changes associated with awareness (Godwin et al., 2015) and similarly, fluctuations of spontaneous activity were demonstrated to correlate with connectivity differences in resting state networks (Scheinost et al., 2016). Network state fluctuations thus, have the potential to explain variance in neural and behavioral responses, specifically regarding measures of task performance, response latencies and neuronal gain, and thereby can enhance the reliability and predictability of the brain’s processing of information (McGinley et al., 2015b).

Network states can be electrophysiologically measured and are highly correlated with changes in pupil diameter (Reimer et al., 2014; McGinley et al., 2015a; Vinck et al., 2015; Reimer et al., 2016), since both, network states and pupil diameter, are controlled by the release of acetylcholine (ACh) and noradrenalin (NA; Aston-Jones and Cohen, 2005; Goard and Dan, 2009; Reimer et al., 2016). A recent demonstration that pupillometry reflects fluctuations of spontaneous neuronal activity and correlates, similar to the network state variable, with sensory evoked responses and performance measures (McGinley et al., 2015a) provides crucial evidence for the association between network state and behavioral outcome in the awake rodent brain. This was found to be the case similarly for measures of intracellular calcium of two-photon imaging data in awake mice. Lu et al. (2017) showed that a substantial fraction of neurons exhibited calcium transients which were strongly correlated with the pupil size of the animal. This positive correlation was particularly consistent for vasoactive intestinal peptide interneurons, a specialized cell class potentially serving to facilitate increases in cortical activity (Jackson et al., 2016) and heightened attention (Reimer et al., 2014). Thus, pupil diameter appears to be a reliable index for underlying network state, which would have a direct application for studies where pupillometry is routinely recorded and direct network state readouts remain challenging.

Physiologically, the correlation of pupil diameter with heart rate and galvanic skin reflex (Tursky et al., 1969) indicates its tight coupling to the peripheral nervous system. Pupil diameter is defined by the interaction of two muscles: the iris sphincter, which receives parasympathetic innervation mainly through cholinergic transmission, controls pupil constriction; and the radial muscle of iris, which receives NA releasing sympathetic fibers, commanding pupil dilation. Recently it was shown that pupil dilation and cortical network state fluctuations may be directly linked through their control by cholinergic and adrenergic projections (Reimer et al., 2016). Besides adapting to changes in luminance, spontaneous pupil diameter changes have been shown to be correlated with changes in arousal, attention, and perception (Einhäuser et al., 2008; Gilzenrat et al., 2010; Murphy et al., 2011, 2014b).

**Figure 1.**
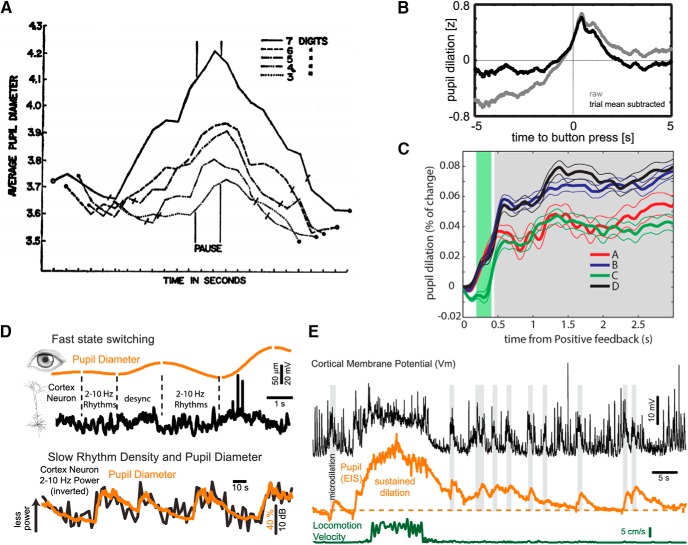
Pupil diameter in human cognitive neuroscience and as an accurate predictor of rapid variations in parameters related to brain state and arousal in the mouse model. ***A***, In a short-memory task, pupil dilation was modulated by the amount of items under active processing at any time: average pupil diameter (mm) for five subjects during auditory presentation (before “pause” period) and recall (after pause period) of digit strings of varying lengths (three to seven digits). The authors found that the pupil dilates when items are presented and constricts during report. The rate of change of these functions was related to task difficulty. From Kahneman and Beatty (1966). ***B***. Pupil dilation departs from baseline ∼1 s before a volitional button press, peaks at 420 ms after the response, and relaxes back to baseline after ∼2 s, thus revealing the time of decision making. Authors interpreted pupil dilation as a marker of NE release from LC and as evidence for the latter role in consolidation of cognitive decisions. From Einhäuser et al. (2010). ***C***, Pupil dilation displays an anticipatory response to uncertainty levels associated with options in a strategic gambling task (Iowa Gambling Task, IGT) where subjects are asked to maximize their profits by choosing the best drawing strategy from a set of four card decks, each of them delivering gains and losses following a pattern unknown for participants. Greater pupil dilation was observed in conditions with a low probability of incoming negative feedback (NF), as compared to conditions where NF had an enhanced probability to occur. Authors interpreted these results as evidence of pupil dilation signaling LC response to decision making in unfamiliar contexts. From Lavín et al. (2014). All axes in ***A–C*** depict pupil dilation (*y*-axis) versus time (*x*-axis). ***D***, upper panel, Simultaneous recording of pupil diameter and membrane potential of a cortical neuron in layer 2/3 of the mouse primary visual cortex. Pupil diameter exhibits spontaneous variations in size even in the synchronized state and in the absence of locomotion. Note the strong relationship between slow (2–10 Hz) rhythmic synaptic activity and constriction, and the suppression of this activity with dilation. Lower panel, Comparison of pupil diameter and density of low-frequency (<10 Hz) rhythmic synaptic activity in a layer 5 pyramidal cell in the auditory cortex. Increases in pupil diameter are associated with prominent suppression of low-frequency synaptic activity (desynchronized state). From McGinley et al. (2015b). ***E***, Whole-cell recordings from a layer 5 pyramidal neuron in auditory cortex of an awake mouse while simultaneously monitoring pupil diameter and locomotion. Brief dilations of the pupil (microdilations), are highlighted in gray and are associated with a suppression of the low-frequency activity and a depolarization of this neuron independent of locomotion. From McGinley et al. (2015b).

In cognitive neuroscience, pupil diameter has additionally been reported to correlate with mental effort in decision-making tasks (Kahneman and Beatty, 1966; Fiedler and Glöckner, 2012; de Gee et al., 2014; Murphy et al., 2014a), as well as with learning dynamics (Nassar et al., 2012; Lavín et al., 2014). Pupil diameter changes on manipulations of task-effort (Fig. 1*A*; Kahneman and Beatty, 1966), with timing of mental decisions (Fig. 1*B*; Einhäuser et al., 2010), perceptual changes as in binocular (Einhäuser et al., 2008) or interauricular (Kang and Wheatley 2015) rivalry, as well as with surprise (Preuschoff et al., 2011) or uncertainty in decision-making tasks (Fig. 1*C*; Lavín et al., 2014; Geng et al., 2015). Although very recently pupillometry has been employed to follow changes in tonic alertness during rs-fMRI experiments (Schneider et al., 2016), pupil dilation as a direct measure for underlying neuronal excitability states has not yet been considered routinely in human cognitive neuroscience.

In this review, we propose that using pupillometry measures as an index for network state fluctuations in humans may be a useful approach to explain unaccounted variance in macroscopic neural and behavioral signals.

## Different Types of Network States

Alternating states of excitability in the waking brain have first been observed in intracellular and local field potential (LFP) recordings of awake rodents. They show large low-frequency fluctuations during periods of quiet resting (Crochet and Petersen, 2006; Poulet et al., 2012; McGinley et al., 2015a). The initiation of whisking or locomotion suppresses the recurrent, slow (<10 Hz) component of the LFP and increases the power of higher frequency oscillations (Poulet et al., 2012; Eggermann et al., 2014; McGinley et al., 2015a). Based on these observations, a classification distinguishing states within waking periods emerged: synchronized states show bimodal, slow rhythmic network activity, and desynchronized states show unimodal persistent, fast network activity. Since network states are continuous and transient phenomena, rather than discrete, categorical conditions, assigning such a dichotomous classification may appear overly simplistic. Nevertheless, classifying data for these substates of waking could explain neuronal response variability and behaviorally relevant correlates in rodents (McGinley et al., 2015a), highlighting its applicability. While the bimodal, synchronized activity was usually related to resting (but see Hall et al., 2014), inattentive behavior, and slower neuronal responses, the persistent desynchronized activity was demonstrated to occur during locomotion or task engagement, showing faster and temporally more precise neuronal responses (Crochet and Petersen, 2006; Pachitariu et al., 2014; McGinley et al., 2015a; Schwalm et al., 2017). In humans, intracortical electrode recordings in the hippocampus of awake subjects undergoing surgical treatment for refractory epilepsy showed similar results: during resting states, slow ripples appeared coordinated in hippocampal areas, whereas in active states during cognitively demanding tasks, high frequency activity emerged in hippocampus and parahippocampal cortex (Billeke et al., 2017). Macroscopic measures similarly demonstrated fluctuating substates in awake human brain activity, during rest (Scheinost et al., 2016; Custo et al., 2017) or during task engagement (Coon et al., 2016; Godwin et al., 2015).

## Pupil Diameter as a Reliable Proxy Marker for Network State

The mentioned network substates, synchronized versus desynchronized activity, can only directly be revealed by invasive readouts reflecting synaptic activity (LFP or subdural electrode array recordings), action potentials (intracellular electrophysiological or calcium recordings), or membrane potentials (whole-cell recordings). The recent introduction of pupil diameter as a reliable peripheral marker for network state fluctuations (Reimer et al., 2014, 2016; McGinley et al., 2015a,b) would allow for classification of network states by means of a noninvasive measure. These studies demonstrated a direct correlation of pupil diameter changes and alterations of intracellular membrane and LFP recordings from cortical and hippocampal neurons in the awake, head-fixed mouse (Fig. 1*D*,*E*). Importantly, in these experiments, pupil diameter has proven to be remarkably accurate in following state changes of cortical network activity, even in the absence of locomotion or other types of movement (Fig. 1*E*; Reimer et al., 2014). Pupil dilation was found to be associated with increases of cortical activation and suppression of slow waves, while pupillary constriction was related to an increase of cortical low-frequency activity. Additionally, microdilations of the pupil were associated with the initiation of brief cortical up states (Fig. 1*E*). The generality of this effect was emphasized by the finding that pupil diameter was correlated to the rate of hippocampal sharp-wave ripples (McGinley et al., 2015a). Furthermore, in humans, pupil dilation can predict fluctuations of fMRI network structures, a measure for global state change (Shine et al., 2016).

As neuronal activity patterns can be highly conserved across species (Buzsáki et al., 2013; Sanchez-Vives et al., 2017) and the neurophysiology of parasympathetic and sympathetic control of pupil dilation seems to be similar in rodents and humans, it is highly likely that the direct relationship between network state and pupil dilation likewise exists in the human brain.

## Physiology of Neuromodulation, Pupil Dilation, and Network State Fluctuations

Neuromodulatory pathways, especially the central cholinergic and noradrenergic pathways, have been shown to be directly involved in shifts of ongoing cortical activity and network responsiveness (Lee and Dan, 2012; Constantinople and Bruno, 2011; Castro-Alamancos and Gulati, 2014; Eggermann et al., 2014; Chen et al., 2015). Importantly, ACh released by the basal forebrain and NA released by the locus coeruleus (LC) are the same neurotransmitters used by the sympathetic and parasympathetic pathways to control pupil diameter (McGinley et al., 2015b). ACh is linked to vigilance and attention and transient prefrontal ACh release can control detection of behaviorally relevant sensory stimuli on multiple time scales (Parikh et al., 2007). Furthermore, the discharge of both basal forebrain cholinergic and LC noradrenergic neurons is increased by attention to external stimuli, arousal, and locomotion (Aston-Jones and Cohen, 2005; Eggermann et al., 2014). The role of the LC has been confirmed in attentional modulation and the regulation of goal-directed versus exploratory behaviors (Usher et al., 1999). LC stimulation can mimic the effects associated with arousal, such as increased amplitudes and precision of sensory evoked responses, as well as increased learning-induced plasticity (Martins and Froemke, 2015; McGinley et al., 2015b) and the discharge of LC neurons is tightly locked to pupil dilation (Aston-Jones and Cohen, 2005; Varazzani et al., 2015).

LC activation is robustly associated with pupil dilation dynamics. Indeed, LC firing reliably anticipates spontaneously occurring, or stimulus-triggered, changes in pupil diameter (Joshi et al., 2016). Likewise, BOLD activity in human LC covaries with pupil dilation (Murphy et al., 2014a). The LC responds to the outcome of task-related decision processes (Clayton et al., 2004), as it also receives synaptic input from the frontal cortex that may be involved in arousal responses to stimuli requiring high-level cognition (Lee and Dan, 2012). The effect of LC firing on cortical state dynamics has been linked to single-trial sensory processing, revealing that the temporal structure of noradrenergic modulation may selectively and dynamically enhance cortical stimulus responses. This suggests a coupling between LC and cortex, that can amplify low-frequency fluctuations and thereby enhance cortical responses to stimuli by tightly timed, phasic LC bursts which account for state-dependent, trial-to-trial variability (Safaai et al., 2015). Recently, the physiologic processes linking network states and pupil variations have been directly linked to activity in noradrenergic and cholinergic projections to the cortex. While long-lasting pupil dilations, such as during locomotion or task engagement, are accompanied by sustained cholinergic activity, brief dilations of the pupil are associated with phasic noradrenergic activity (Reimer et al., 2016).

## Network State Fluctuations Underpin Arousal and Performance

The inverted-U relationship between arousal and performance (percentage correct, latency, discriminability) proposed by Yerkes and Dodson (1908) situates optimal behavioral performance at intermediate levels of arousal, with extremes of low and high arousal leading to poor performance, due to disengagement and exhaustion respectively. Performance peaks at intermediate arousal levels, particularly in behavioral tasks involving high-level, prefrontal cortex-dependent cognition. This relationship depends on the activation level of receptors for the aforementioned neuromodulators, which are also involved in pupil diameter changes.

The inverted-U relationship was confirmed between task performance in a go/no-go task and neuronal activity in the mouse auditory cortex (McGinley et al., 2015a). Moreover, this relationship held true for correct behavioral responses, pupil diameter, and evoked neuronal responses. Hit rates and neuronal gain were highest and response latencies shortest at intermediate pupil diameters. In macaques, the ability to perform a delayed saccade to target task was found to be optimal at intermediate pupil diameters (Ebitz et al., 2014). In humans, variations in spontaneous activity have similarly been associated with changes in sensory perception and task performance (Schroeder and Lakatos, 2009) as revealed by fMRI (Boly et al., 2007), electroencephalography (EEG; Hesselmann et al., 2008), and magnetoencephalography (MEG; Baumgarten et al., 2015). Furthermore, Murphy et al. (2011) showed response latency for correct stimulus detection in an auditory oddball task to be shortest, and P3 potential in EEG to be maximal, at medium pupil diameters.

Thus, variations in the arousal of awake human subjects contain an optimal zone for neuronal and behavioral responses which can be determined by tracking cortical state fluctuations either directly, through electrophysiological recordings, or indirectly, by using pupil dilation measurements. Moreover, network states have been shown to influence sensory processing efficiency and response times and might further extend to higher-order effects on learning and memory-related tasks.

Concerning stimulus-response properties, during the desynchronized state, mouse barrel cortex neurons show a lower stimulus detection threshold, higher response fidelity, and shorter response latency (Fazlali et al., 2016). In auditory cortex, noise correlations decrease and neuronal responses to tones become temporally more precise and reliable during the desynchronized state (Pachitariu et al., 2014). This supports the notion of desynchronized activity being required to accurately process sensory information (McGinley et al., 2015b; Kaneko et al., 2017).

Since human behavioral response timing is highly variable from trial to trial, including the timing of neuronal activity as a variable would be helpful to explain this variability. Tracking neuronal population activity across the human cortex demonstrated how variations in the timing of neural activity relate to variations in the timing of behavior in a modified Posner visual-attention cueing task (Posner 1980; Coon et al., 2016). Whereas the authors interpreted their results in terms of oscillatory phase modulation of the information’s transmission speed in cortical networks, and its resulting behavior, they critically noticed a broadband activity surge preceding behavioral responses, which can be interpreted as a change in network state. In rs-fMRI data, it was shown that the human brain alternates between functional states, linking cognitive performance and the dynamic reorganization of the network structure reflected by pupil dilation (Shine et al., 2016). Similar to the data obtained in rodents (Reimer et al., 2014; McGinley et al., 2015a), in this study, faster and more effective cognitive performance (measured as shorter nondecision time during an N-back task) was related to increases in pupil diameter.

Putting these previous findings into perspective regarding underlying neural activity, it seems worthwhile exploring such datasets while employing pupil diameter as a direct measure for network state. As there is already work showing the influence of network state on basic behavioral response probabilities and preliminary evidence for a role in higher cognitive function, we propose to explore the influences of network state indexed by pupil dilation on cognitive and decision-making tasks.

## Network States and Pupil Dilation in Decision-Making Tasks

Pupil diameter has been used as a proxy measure for arousal in effortful decision-making tasks (Kahneman and Beatty, 1966; Fiedler and Glöckner, 2012; de Gee et al., 2014; Murphy et al., 2014a). Changes of pupil dilation have been investigated in a financial-choice paradigm showing sustained pupil dilation throughout decision formation (Fiedler and Glöckner, 2012). Task-independent shifts of arousal state, indicated by enhanced pupil diameter, have been related to higher trial-by-trial variability in the rate of evidence accumulation during perceptual decision making (Murphy et al., 2014b). Along the same lines, pupil dilation has been linked to cognitive variables influencing the decision-making process (Gilzenrat et al., 2010; Preuschoff et al., 2011; Cavanagh et al., 2014). Such decision processes involve the calculation of unexpected uncertainty associated with LC-NA activity (Aston-Jones and Cohen, 2005; Yu and Dayan, 2005; Lavín et al., 2014; Geng et al., 2015).

Although it has been proposed that changes in network state interact with biased decision making in the face of uncertainty (de Gee et al., 2014), the relationship between pupil dilation and decision-making processes has previously been studied from the perspective of task-evoked changes in pupil diameter. From this perspective, environmental changes (e.g. stimuli signaling uncertainty) are assumed to trigger a change in LC firing mode which leads to a release of ACh and NA, which in turn induce a change of pupil diameter. Whereas the association between pupil dilation and arousal state has been indirectly assumed in many decision-making studies (Nassar et al., 2012; de Gee et al., 2014; Murphy et al., 2014b), until now a direct link between task-independent brain state fluctuations and behavioral performance and learning in decision making has not been sufficiently investigated. Also, in the absence of environmental stimulation, global changes in brain state can lead to an alteration of LC firing and neuromodulator levels, which are reflected in pupil diameter change but also have their correlate in differential neuronal responses and differential behavioral outcome.

As explained above, pupil dilation has routinely been measured in decision-making studies and was also related to cognitive variables, such as arousal, and to neuromodulatory fluctuations. However, the finding of pupil dilation directly reflecting underlying excitatory states of the neural network opens another dimension of explanatory value. Introducing a network-state related variable and separating behavioral and neural data based on pupil dilation measures would, through *post hoc*, dedicated analyses, help explain previously unaccounted variance in decision-making studies. Hence, this approach could provide a novel, neurophysiologically defined technique of exploring the underpinnings of everyday choices.

## Considering Pupil-Indexed Network State as a Variable in Cognitive Neuroscience

Considering the well-established physiologic evidence outlined above, internal brain dynamics which have previously been shown to underlie neuronal responses may similarly influence high-level cognition, decision-making outcomes and learning. This influence on high-level brain functions has already been explored in rodents and primates (Harris and Thiele, 2011), the timing of LC activity has been shown to track behavioral responses more closely than stimulus presentation (Clayton et al., 2004), and LC firing has an effect on cortical state (Safaai et al., 2015). However, evidence linking pupil-indexed network state to performance and learning in humans has not been reported., Hence, it is reasonable to use physiologic measures of underlying network state fluctuations, such as LC-controlled pupil dilation (Gilzenrat et al., 2010; Murphy et al., 2014b), to predict performance and learning in cognitive tasks in humans.

First steps in the exploration of learning dynamics regulated by pupil-linked arousal systems have already been made: brief changes in pupil dilation predicted the reliability of responses in a predictive inference task (Nassar et al., 2012). These authors also predicted task-independent manipulations of pupil diameter to alter behavior, since higher learning rates were associated with small baseline pupil diameter and lower learning rates were associated with large baseline diameter. This finding is consistent with the hypothesis that larger pupil diameters are associated with desynchronized states of the network and smaller pupil diameter indicate underlying synchronized states (McGinley et al., 2015a,b).

## Implementing Pupil-Indexed Network State Measures by Pupillometry and Data Analysis

Establishing a link between spontaneous network fluctuations, pupil diameter, and behavioral performance is achievable in experiments recording whole-brain activity. Macroscopic measures (EEG, MEG, or fMRI) can be related to pupillometry, and to behavioral performance, learning, or decision-making outcomes.

It is well established that pupil diameter fluctuates in response to changes in environmental luminance. Thus, the first step in any study aiming to record meaningful pupillometry data are to ensure that all visual stimulation is isoluminant and no sudden transitions in visual stimulation are included in the experimental paradigm, since these may induce pupillary responses triggered by visual novelty. Additionally, subjects should be screened for preserved central and peripheral innervation to pupil muscles (i.e., consensual response in photopupillary reflex).

The recording of pupillometry is achieved noninvasively by camera-based, infrared eyetrackers. Commercial alternatives are matched by open-source systems, which in some cases include their own analysis software (Zimmermann et al., 2016) making them particularly user friendly and adaptable to the desired experimental paradigm. Usually, the pupil area or diameter is reported in arbitrary units. The time series obtained from the eyetracker system can be z-transformed, and missing data caused by blink periods can be interpolated using linear or spline methods.

The recorded pupillometry datasets can be sorted offline for sustained dilation or constriction periods, indicative of desynchronized (“active”) and synchronized (“resting”) network states respectively. Here, exact thresholds for pupil dilation, microdilation and constriction remain to be established, but tracking the first derivative of pupil area would allow for detection of rapid changes based on the signal's slope. Once sorted, the data (e.g., EEG signals) recorded during putative desynchronized or synchronized states can be analyzed accordingly and response times or learning and decision-making parameters can be compared between states. Specific data analysis techniques which would be appropriate include linear regression or representational similarity analysis (Kriegeskorte et al., 2008), among a plethora of approaches aiming to predict the neuronal or the behavioral signal on the network state periods previously classified by pupil dilation. With this approach, also for the human brain, potential network state influences can be revealed and previously unaccounted variance in the ongoing signal or in trial-to-trial variability might be explained.

## Conclusion

We encourage employing pupillometry as a noninvasive, robust proxy measure of ongoing network states and highlight the need to verify the corresponding predictions derived from animal models in humans.

Fluctuations between cortical states occur constantly and frequently throughout awake periods (Buzsáki, 2006; Poulet and Petersen, 2008; McGinley et al., 2015a). These continuous changes can contribute to the variability of experimental results in awake recordings and may appear as noise if they are disregarded in the statistical modeling of neural signals. Including a fine-grained classification of network state as independent variable (i.e., covariate, regressor, or entirely new factor) could explain a considerable portion of variance in neuronal and behavioral responses, resulting in an increase of signal-to-noise ratio and more reliable readouts of human brain activity. Hence, considering network state fluctuations opens the possibility to explore and predict neural mechanisms of behavior, sensory coding, decision-making and motor responses more accurately and eventually reveal trial-to-trial variability to be more predictable than previously thought.

Investigating the direct interrelation of underlying global EEG, MEG, or fMRI activity reflecting fluctuations of brain state and pupil diameter changes, and relating both measures to behavioral performance, learning and decision making, would put previous findings into perspective, eventually demonstrating a dependency of higher-order cognitive operations on internally generated states of the brain. Spontaneous fluctuations of ongoing neuronal activity likely influence behavioral outcomes and learning, but can equally be altered by environmental stimulation, and thus represent an important mediator variable which should be considered in a broad range of neurophysiological measurements. The possibility to track these state changes within human cognitive experiments by pupil dilation measures could greatly enhance the current knowledge about the dependency of human decision-making processes on neural network states.

The work reviewed here suggests a direct relationship of changes in pupil diameter and cortical state fluctuations in the awake brain. As discussed above, pupil diameter has been commonly associated with psychological constructs, notably arousal and cognitive effort, where a consistent relationship with NA neurons in LC has gained ample empirical support. However, until recently, the close, phase-locked relationship with brain state reflected by membrane and local LFPs was not known. The question remains whether pupil dilation also proves as a reliable biomarker for the described substates of wakefulness in human subjects, and whether it can be used to predict neuronal responses, task performance and learning, beyond sensory perception paradigms. It is plausible that results in humans will follow those in animal models, given how conserved state variations are across mammalian brains (Buzsáki et al., 2013). Ultimately, the identification of a direct, unequivocal brain signature could lead to the refinement of constructs such as arousal or cognitive effort in favor of a precise neurophysiological variable provided by a pupil dilation-defined network state prediction.
